# Association Between Serum Cotinine Level and Serological Markers of Epstein–Barr Virus in Healthy Subjects in South China Where Nasopharyngeal Carcinoma Is Endemic

**DOI:** 10.3389/fonc.2019.00865

**Published:** 2019-09-13

**Authors:** Qian-Yi Yang, Yong-Qiao He, Wen-Qiong Xue, Ting Zhou, Ying Liao, Mei-Qi Zheng, Yi-Jing Jia, Lei-Lei Yuan, Wei-Hua Jia

**Affiliations:** ^1^State Key Laboratory of Oncology in South China, Collaborative Innovation Center for Cancer Medicine, Guangdong Key Laboratory of Nasopharyngeal Carcinoma Diagnosis and Therapy, Sun Yat-sen University Cancer Center, Guangzhou, China; ^2^Department of Anesthesiology, Sun Yat-sen University Cancer Center, Guangzhou, China; ^3^School of Public Health, Sun Yat-sen University, Guangzhou, China; ^4^Cancer Center of Guangzhou Medical University, Guangzhou, China

**Keywords:** Epstein–Barr virus (EBV), nasopharyngeal carcinoma (NPC), smoking, VCA-IgA, cotinine, Chinese

## Abstract

**Introduction:** Self-reported smoking has been associated with higher seropositivity for the IgA response to Epstein–Barr virus (EBV) viral capsid antigen (VCA-IgA) and transcription activator protein (Zta) in healthy men in southern China where nasopharyngeal carcinoma (NPC) is endemic. Results on the association of biochemically verified smoking status with EBV reactivation are scarce. We aimed to investigate the relations of serum cotinine level with serological markers of EBV in healthy women, in addition to men.

**Methods:** We collected information on demographic, lifestyle, environmental factors, and EBV serological markers in a cross-sectional study on 2,275 healthy subjects who were recruited from physical examination centers in Guangdong Province, China. In the present analysis, 901 subjects' serum cotinine levels have been measured by enzyme-linked immunosorbent assay (ELISA). Odds ratios (seropositivity of four EBV serological markers vs. seronegativity) for categorical serum cotinine levels were calculated by unconditional logistic regression with a group-specific confidence interval (CI).

**Results:** In women, compared with lower serum cotinine level (0–0.71 ng/ml), higher cotinine level (>0.71–1.20 ng/ml; >1.20–228.40 ng/ml) was associated but non-significantly with higher seropositivity for EBV VCA-IgA (age- and education-adjusted OR = 1.18, 95% CIs = 0.84–1.64, 1.06, 0.75–1.50). These associations remained but still non-significant after adjusting for 5-year age group, education, family history of cancer, consumption of tea, Chinese herbal tea, salted fish at childhood, and exposure to occupational dust, chemical, fume, and radiation (multivariable adjusted OR = 1.21, 95% CIs = 0.85–1.71, 1.09, 0.76–1.55). In men, compared with lower serum cotinine level (0–2.15 ng/ml), higher cotinine level (>2.15–103.6 ng/ml; >103.6–419.4 ng/ml) was significantly associated with higher seropositivity for EBV VCA-IgA and Zta-IgA (age- and education-adjusted OR = 2.16, 95% CIs = 1.37–3.41, 1.79, 1.11–2.90; 1.98, 1.17–3.34, 1.95, 1.14–3.34). The association remained significant after adjusting for potential confounders for Zta-IgA (OR = 2.32, 95% CI = 1.37–3.93 for >2.15–103.6, and 2.50, 1.43–4.38 for >103.6–419.4 ng/ml), but not for VCA-IgA (2.06, 1.29–3.27, and 1.61, 0.96–2.71).

**Conclusions:** Higher serum cotinine level is associated with higher seropositivity for EBV serological markers in healthy men in southern China. Such positive association was also observed in women but became non-significant. If confirmed to be causal, this finding has important implications for tobacco control and prevention of EBV-related disease, particularly for NPC.

## Introduction

Epstein–Barr virus (EBV) is a ubiquitous human herpesvirus, infecting over 90% of the adult population worldwide (1). EBV has been rated as a Group 1 carcinogen by the International Agency for Research on Cancer, because of its association with nasopharyngeal carcinoma (NPC) (2). Primary EBV infection in developing countries usually occurs early in life, and most cases sustain an asymptomatic and lifelong infection (3). EBV infects and then persists latently in nearly all humans. However, NPC remains very rare in the world, except southern China, suggesting reactivation of EBV is necessary in the pathogenesis of NPC (4). The inducers of EBV reactivation are largely unclear. Our previous study has first reported that self-reported smoking initiation at younger age and higher cumulative amount of smoking (vs. never smoking) were associated with higher seropositivity for the IgA response to viral capsid antigen (VCA-IgA) in healthy men in Guangdong province, southern China where NPC is endemic (5). Furthermore, our recent study has also shown that self-reported smoking was associated with higher levels of other EBV antibodies (EBNA1-IgA and BZLF1 transcription activator protein [Zta-IgA]) in the same population (6). Results on the association between smoking and EBV reactivation in women are scarce, probably because of low smoking prevalence (about 3.4%) and a large intergenerational decrease in smoking uptake rates in Chinese women (7, 8). The validity of self-reported smoking is often questioned because of the common belief that smokers are inclined to underestimate the amount smoked or to deny smoking at all (9), particularly in women in China where smoking is usually socially undesirable (10).

An increasing number of studies have used biomarkers in assessing smoking exposure, which improve the validity of self-report smoking. Biomarkers can be used to classify people as smoking exposed or unexposed, identify deceivers (i.e., subjects misreporting their smoking status), or estimate relative degree of exposure. Cotinine, a major metabolite of nicotine, has been regarded as a gold standard of biomarker of smoking, and can provide objective data of smoking exposure.

We thereby examined the associations of smoking status (classified by serum cotinine level) with four serological markers of EBV (VCA-IgA, Zta, EBNA1, and latent membrane protein 1 [LMP1]) in healthy men that can validate our results with the previous studies, and first investigated the association of serum cotinine level with EBV VCA-IgA serology in healthy women.

## Methods

This study was part of the large-scale NPC case–control study in Guangdong province, southern China. In the present analysis, we examined the cross-sectional data on 2,275 healthy subjects who were recruited from physical examination centers in the 21-RCCP study (21 regions in Guangdong Province collected from the Cantonese population). Details about the design, methods, and subjects have been described elsewhere (11). Briefly, all the subjects were Chinese without history of cancer. Information on demographic characteristics, occupational exposure, family history of cancer, smoking and drinking status, consumption of salted fish, preserved vegetable, tea and Chinese herbal tea was collected using a face-to-face, structured questionnaire in the physical examination centers. Each subject provided written informed consent for an interview and a 5–10 ml blood sample. All the EBV serological tests were conducted by the same technicians (sample information was blinded) in the same laboratory at Sun Yat-sen University Cancer Center to limit potential biases. The antibody titers of VCA-IgA, Zta-IgA, EBNA1-IgA, and LMP1-IgA were measured as indicators of EBV status using commercial enzyme-linked immunosorbent assay (ELISA) kits (Zhongshan Bio-tech Co Ltd., China). Details of sample collection and processing were reported (5, 12, 13). Briefly, for VCA-IgA in all subjects, serial dilutions of quality control sera (1:10, 1:20, 1:40, 1:80) were applied to each assay for the evaluation of intra-variability, and those subjects with titers ≥1:10 were categorized as positive. In men only, LMP1 peptide was derived from Yeast-expressed EBV strain (GD1) 185–366aa, and EBNA1 and Zta were produced with purified recombinant peptides specified by ENB BKRF1 (72 kDa) and KZLF1 (36 + 38-kDa fine doublet), respectively. The serostatus of LMP1-IgA, EBNA1-IgA, and Zta-IgA was defined as seronegative or seropositive according to the ELISA OD values following the manufacturers' instruction. For EBNA1-IgA, a weak seropositive status defined by the manufacturer's instruction was classified as seropositive in the present analysis. Due to the limited resources, all female subjects (*N* = 665) were enrolled, 300 male subjects were stratified sampling based on 5-year age group. Fifty-eight female and six male subjects were excluded from the analysis because of insufficient blood samples for additional cotinine test. In total, 607 female and 294 male subjects were included in the present analysis. Serum cotinine level was measured using a commercial immune-analysis cotinine ELISA kit provided by R&D Systems, and the results were expressed as ng/ml (sensitivity: 0.001 ng/ml).

## Statistical Analysis

Generalized linear model with binomial distribution was used to calculate odds ratios (ORs) of EBV VCA-IgA (seropositive vs. seronegative) for the about tertile of serum cotinine levels stratified by sex with/without adjusting for 5-year age group, education, family history of cancer, salted fish at adulthood, and exposure to any occupational hazards (dust, chemical, fume, or radiation). We estimated 95% confidence intervals (CIs) for each group (including the reference) that corresponded to the amount of information underlying each group, using Plummer's methods (14), in the variable with three or more groups. To assess dose–response effect, a *P*-value for linear trend was determined by treating the exposure variables as continuous. Interactions or effect modifications were explored by including the interaction terms in the models. No evidence of interaction (in the model adjusting for age and education) by family history of cancer, smoking (no ever smokers), alcohol, consumption of salted fish, and exposure to any occupational hazards was found for the association between serum cotinine level and serostatus of different EBV markers (*P*-values for interaction ranged from 0.11 to 0.99 in women, and 0.15–0.97). Statistical analyses were done with R 3.5.1, and all tests were two-sided with α = 0.05.

## Results

Table 1 shows that differences in education level (*P* < 0.001), smoking (*P* < 0.001), drinking (*P* < 0.001), consumption of salted fish at adulthood (*P* = 0.003), and exposure to any occupational hazards (all *P*-values: <0.01) were observed between men and women. Very few women were ever smokers (1.5%), but 72.8% of men ever smoked (58 ex-smokers and 156 current smokers). 78.1% of women were never drinkers comparing with 33.7% of never drinkers in men. Men were more likely to be exposed to any occupational hazards (dust: 58.5%, chemical: 52.4%, fume: 40.1%, or radiation: 10.5%). In women, the corresponding figure for exposure to any occupational hazards was 39.0, 27.0, 9.1, and 5.8%. No difference in age, family history of cancer, and consumption of salted fish at age 10 was observed (all *P*-values >0.05).

**Table 1:** Demographic characteristics of the study populations in 607 women and 294 men.

**Variables**	**Women (*N*)**	**(%)**	**Men (*N*)**	**(%)**	***P*-value^‡^**
**Age, mean (SD)**	44.68 (11.6)		45.98 (11.5)		0.11
18–29	51	8.4	15	5.1	0.13
30–49	354	58.3	165	56.1	
50–64	172	28.3	93	31.6	
65+	30	4.9	21	7.1	
**Education**					
Primary or less	122	20.1	13	4.4	<0.001
Middle school	317	52.2	192	65.3	
University or more	167	27.5	88	29.9	
Missing	1	0.2	1	0.3	
**Family history of cancer**					
No	416	68.5	187	63.6	0.13
Yes	174	28.7	92	31.3	
Missing	17	2.8	15	5.1	
**Smoking**					
Never	598	98.5	80	27.2	<0.001
Ex-	3	0.5	58	19.7	
Current	6	1.0	156	53.1	
**Consumption of alcohol**					
Never	474	78.1	99	33.7	<0.001
≤1 drink/day	127	20.9	150	51.0	
>1 drink/day	6	1.0	44	15.0	
Missing	0	−	1	0.3	
**Salted fish**					
Childhood (at age 10)					0.52
Less than monthly	464	76.4	224	76.2	
Monthly	48	7.9	30	10.2	
Weekly or more	86	14.2	38	12.9	
Missing	9	1.5	2	0.7	
Adulthood (at recruitment)					0.003
Less than monthly	529	87.1	278	94.6	
Monthly	37	6.1	11	3.7	
Weekly or more	33	5.4	4	1.4	
Missing	8	1.3	1	0.3	
**Occupational exposure**					
Dust					<0.001
Unexposed	370	61.0	121	41.2	
Exposed	237	39.0	172	58.5	
Missing	0	−	1	0.3	
Chemical					<0.001
Unexposed	443	73.0	139	47.3	
Exposed	164	27.0	154	52.4	
Missing	0	−	1	0.3	
Fume					<0.001
Unexposed	552	90.9	175	59.5	
Exposed	55	9.1	118	40.1	
Missing	0	−	1	0.3	
Radiation					0.007
Unexposed	572	94.2	262	89.1	
Exposed	35	5.8	31	10.5	
Missing	0	−	1	0.3	

‡ *t-test and chi-square test were used to compare the mean of continuous factors and proportions of categorical factors between cases and controls, respectively*.

Table 2 shows that in women, the multivariable ORs (95% CIs) of being seropositive (vs. seronegative) in EBV VCA-IgA for the about tertile of serum cotinine levels (>0.71–1.20 and >1.20–228.40 vs. 0–0.71 ng/ml) were 1.21 (0.85–1.71) and 1.09 (0.76–1.55) (*P*-value for trend = 0.75).

**Table 2:** Odds ratios (ORs) and 95% confidence intervals (CIs) of EBV VCA-IgA antibody titers for serum cotinine level in women.

**Serum cotinine, ng/ml**	**No. of subjects with ±**	**Age- and education-adjusted OR (95% CI)**	**Multivariable^†^ OR (95% CI)**
0–0.71	163/39	1.00 (0.70–1.42)	1.00 (0.69–1.44)
>0.71–1.20	158/44	**1.18** (0.84–1.64)	**1.21** (0.85–1.71)
>1.20–228.40	162/41	**1.06** (0.75–1.50)	**1.09** (0.76–1.55)
*P*-value for trend		0.81	0.75

±: seronegative vs. seropositive.

† Adjusted for age (5-year group), education, family history of cancer, consumption of salted fish at adulthood, and exposure to any occupational hazards (dust, chemical, fume, or radiation).

VCA-IgA, IgA against viral capsid antigen.

*No significant interactions by family history of cancer, smoking (no ever smokers), alcohol, consumption of salted fish, and exposure to any occupational hazards were observed for the association between serum cotinine level and EBV VCA-IgA serostatus (P-values for interaction ranged from 0.11 to 0.99). Bold indicate the OR values*.

Table 3 shows that in men, the age- and education-adjusted ORs (95% CIs) of being seropositive (vs. seronegative) in EBV VCA-IgA for the about tertile of serum cotinine levels (>2.15–103.6 and >103.6–419.4 vs. 0–2.15 ng/ml) were 2.16 (1.37–3.41) and 1.79 (1.11–2.90) (*P*-value for trend = 0.20). The corresponding values in the other three serological markers of EBV (Zta-IgA, EBNA1-IgA, and LMP1-IgA), respectively, were 1.98 (1.17–3.34) and 1.95 (1.14–3.34) (*P*-value for trend = 0.15), 1.53 (0.99–2.38) and 1.33 (0.86–2.05) (0.39), and 1.29 (0.79–2.13) and 1.53 (0.90–2.61) (0.30). These positive associations remained but became non-significant in the multivariable model except for Zta-IgA.

**Table 3:** ORs and 95% CIs of Epstein–Barr virus serological markers (VCA-IgA, Zta-IgA, EBNA1-IgA, and LMP1-IgA) for serum cotinine level in men.

	**VCA-IgA**			**Zta-IgA**			**EBNA1-IgA**			**LMP1-IgA**		
**Serum cotinine, ng/ml**	**No. of subjects with ±**	**Age- and education-adjusted OR (95% CI)**	**Multivariable^†^ OR (95% CI)**	**No. of subjects with ±**	**Age- and education-adjusted OR (95% CI)**	**Multivariable^†^ OR (95% CI)**	**No. of subjects with ±**	**Age- and education-adjusted OR (95% CI)**	**Multivariable^†^ OR (95% CI)**	**No. of subjects with ±**	**Age- and education-adjusted OR (95% CI)**	**Multivariable^†^ OR (95% CI)**
0–2.15	86/12	1.00 (0.53–1.88)	1.00 (0.52–1.93)	84/10	1.00 (0.50–2.00)	1.00 (0.47–2.15)	58/8	1.00 (0.64–1.56)	1.00 (0.62–1.62)	50/16	1.00 (0.55–1.81)	1.00 (0.54–1.86)
>2.15–103.6	72/25	**2.16** (1.37–3.41)	**2.06** (1.29–3.27)	75/17	**1.98** (1.17–3.34)	**2.32** (1.37–3.93)	58/17	**1.53** (0.99–2.38)	**1.43** (0.91–2.23)	54/21	**1.29** (0.79–2.13)	**1.20** (0.72–1.99)
>103.6–419.4	77/22	**1.79** (1.11–2.90)	**1.61** (0.96–2.71)	80/17	**1.95** (1.14–3.34)	**2.50** (1.43–4.38)	60/10	**1.33** (0.86–2.05)	**1.60** (0.98–2.59)	49/22	**1.53** (0.90–2.61)	**1.42** (0.81–2.49)
*P*-value for trend		0.20	0.33		0.15	0.07		0.39	0.18		0.30	0.41

±Seronegative vs. seropositive.

† Adjusted for 5-year age group, education, family history of cancer, consumption of salted fish at adulthood, and exposure to any occupational hazards (dust, chemical, fume, or radiation).

VCA-IgA, IgA against viral capsid antigen; Zta-IgA, IgA against BZLF1 transcription activator protein; EBNA1-IgA, IgA against Epstein–Barr virus nuclear antigen 1; LMP1-IgA, IgA against latent membrane protein 1.

*No significant interactions by family history of cancer, smoking, alcohol, consumption of salted fish and exposure to any occupational hazards were observed for the associations between serum cotinine level and serostatus of different EBV markers (P-values for interaction ranged from 0.15 to 0.97). Bold indicate the OR values*.

## Discussion

A landmark report by the International Agency for Research on Cancer in 2014 stated that NPC is considered to be causally related to tobacco smoking. Such association was mainly observed in low-risk regions for squamous cell NPC, but was rarely reported for non-keratinizing NPC, which is the predominant histology subtype in high-risk regions of NPC, probably due to the different stages of tobacco epidemic between developed (advanced) and developing (early) countries/regions (15). Most high-risk regions of NPC are developing countries/regions where tobacco consumption increases substantially in recent years (16). Indeed, since the 2000s, smoking has also been found to be associated with non-keratinizing NPC (17–25).

The present analysis is the first study to investigate the relation between serum cotinine level and EBV serology status of the general population in southern China where NPC is endemic. We found that higher serum cotinine level was associated with higher seropositivity for VCA-IgA and Zta-IgA in men. This finding is consistent with our previous studies (5, 6), suggesting that our data should be robust.

Of the non-smokers in Mainland China, more than 53.5% were passively exposed to tobacco smoke for more than 15 min per day for at least 1 day per week (26). When the numbers of active and passive smokers are combined, more than 72% of them are exposed to tobacco smoke (26). Given this high proportion of passive smoking exposure in Mainland China, inadequate assessment of exposure to passive smoking can contaminate the reference groups of never smoking, thus resulting in underestimating the effect of smoking. Therefore, we first used serum cotinine level to estimate the “real” exposure of smoking. Indeed, we did find that higher serum cotinine level was significantly associated with higher seropositivity for EBV VCA-IgA and Zta-IgA in men.

In addition to the findings in men, the present study is the first to report that in women, higher serum cotinine level was associated but non-significantly with higher EBV VCA-IgA seropositivity in China where female smoking prevalence is very low (2.4% in 2010). Although the present analysis included largest female subjects, our sample size of 607 (statistical power = 54%, assuming 1.45% cotinine-verified ever smokers with EBV VCA-IgA seronegativity and 3.23% with seropositivity, at a significance level of 0.05) would have a 46% probability of failing to detect a significant difference when one does exist (type II error). Further research on a larger sample size (at least 1,129 subjects, with 80% power) is warranted. Indeed, a recent prospective cohort study on 10,181 residents in Sihui city (27), southern China, showed that in non-NPC subjects, ever smokers were associated with higher odds of EBV seropositivity at baseline (VCA-IgA: adjusted OR = 1.40, 95% CI = 1.22–1.60; EBNA1-IgA: 1.58, 1.38–1.82), and in the 3–5 years of follow up (VCA-IgA: 1.68, 1.29–2.18; EBNA1-IgA: 1.92, 1.42–2.59).

EBV reactivation plays an important role of NPC (28), suggesting that understanding the factors related to EBV reactivation is important. Our previous studies have shown smoking, also a Group 1 carcinogen, was associated with EBV reactivation. The present findings in men and in women using biochemically verified smoking status provided additional evidence that smoking (active or passive) is associated with EBV reactivation.

This study has several limitations beyond those previously mentioned. First, our results did not examine the role of passive smoking on EBV serostatus, as information on second hand smoke exposure was not collected. Collecting a comprehensive list of the sources of exposure to passive smoking is warranted. Selection bias among men might have occurred. However, no difference between included and excluded men was found (data not shown), suggesting that the influence would not be large. Furthermore, despite adjusting for several potential confounders, residual confounding cannot be ruled out.

## Conclusions

Our study provided additional evidence to support the idea that higher serum cotinine level is associated with higher seropositivity for EBV serological markers in healthy men in southern China where NPC is endemic. Furthermore, we have also found positive but non-significant associations between higher serum cotinine level and EBV VCA-IgA seropositivity in women. Strong tobacco control measures are needed to prevent subjects with high risk of NPC from the harmful effects of smoking. Smoking cessation also should be advocated in the endemic regions of NPC.

## Data Availability

The data that support the findings of this study are available from the corresponding author upon reasonable request.

## Ethics Statement

The studies involving human participants were reviewed and approved by Human Ethics Approval Committee at Sun Yat-sen University Cancer Center. The participants provided their written informed consent to participate in this study.

## Author Contributions

W-HJ conceived, designed the study, and wrote the final draft. Q-YY participated in the study design, performed the data collection and analysis, drafted the manuscript, and has checked the accuracy and completeness of the references. Y-QH, W-QX, TZ, and YL assisted the experiments. M-QZ, Y-JJ, and L-LY assisted the participant recruitment and data collection. All authors revised the text critically for important intellectual content and contributed to final approval of the paper.

### Conflict of Interest Statement

The authors declare that the research was conducted in the absence of any commercial or financial relationships that could be construed as a potential conflict of interest.
